# Estradiol and high fat diet associate with changes in gut microbiota in female *ob/ob* mice

**DOI:** 10.1038/s41598-019-56723-1

**Published:** 2019-12-27

**Authors:** Kalpana D. Acharya, Xing Gao, Elizabeth P. Bless, Jun Chen, Marc J. Tetel

**Affiliations:** 1 Neuroscience Department, Wellesley College, Wellesley, MA 02481 USA; 2 Department of Health Sciences Research & Center for Individualized Medicine, Mayo Clinic, Rochester, MN 55905 USA

**Keywords:** Endocrine system and metabolic diseases, Microbiome

## Abstract

Estrogens protect against diet-induced obesity in women and female rodents. For example, a lack of estrogens in postmenopausal women is associated with an increased risk of weight gain, cardiovascular diseases, low-grade inflammation, and cancer. Estrogens act with leptin to regulate energy homeostasis in females. Leptin-deficient mice (*ob/ob*) exhibit morbid obesity and insulin resistance. The gut microbiome is also critical in regulating metabolism. The present study investigates whether estrogens and leptin modulate gut microbiota in ovariectomized *ob/ob* (obese) or heterozygote (lean) mice fed high-fat diet (HFD) that received either 17β-Estradiol (E2) or vehicle implants. E2 attenuated weight gain in both genotypes. Moreover, both obesity (*ob/ob* mice) and E2 were associated with reduced gut microbial diversity. *ob/ob* mice exhibited lower species richness than control mice, while E2-treated mice had reduced evenness compared with vehicle mice. Regarding taxa, E2 was associated with an increased abundance of the S24-7 family, while leptin was associated with increases in Coriobacteriaceae, *Clostridium* and *Lactobacillus*. Some taxa were affected by both E2 and leptin, suggesting these hormones alter gut microbiota of HFD-fed female mice. Understanding the role of E2 and leptin in regulating gut microbiota will provide important insights into hormone-dependent metabolic disorders in women.

## Introduction

Estrogens profoundly influence energy homeostasis^1–3^, as well as reproductive physiology and behavior^4–6^. Estrogens reduce food intake, attenuate body weight gain and adiposity, and increase physical activity in humans and rodents^1,2^. Postmenopausal women have lower levels of circulating estrogens and an increased tendency to gain fat weight, which increases their risk for obesity, cardiovascular disease, stroke, and type 2 diabetes^7–9^. Similarly, in mice on a high-fat diet (HFD), ovariectomy increases energy intake and obesity, while estradiol (E2) treatment prevents weight gain^2,10–13^, indicating that estrogens protect against HFD-induced obesity.

Leptin is a peptide hormone secreted primarily by adipocytes, which acts primarily in the brain to stimulate metabolism, promote satiety, and regulate fat storage^14–16^. A mutation in the *ob* gene that encodes leptin results in mice lacking the hormone (*ob/ob)*^17^. While phenotypically normal at birth, *ob/ob* mice quickly develop obesity and diabetes^18^. Additionally, *ob/ob* mice exhibit increased food intake and decreased physical activity, energy metabolism, and body temperature compared to lean controls, making *ob/ob* mice an excellent genetic model of obesity^19–21^. Administering leptin to adult *ob/ob* mice reverses these effects by decreasing food intake, increasing energy output and decreasing circulating levels of glucose and insulin^19,22^.

Leptin and estrogen signaling pathways interact to influence reproduction and energy metabolism. High levels of E2 are associated with increased leptin sensitivity in both male and female rodents^23^. In contrast, decreased estrogens in ovariectomized mice and postmenopausal women increases leptin resistance, resulting in obesity^24,25^. While female *ob/ob* mice do not have an estrous cycle, leptin administration restores fertility, including successful ovulation, pregnancy, and birth, indicating the profound effects of leptin on reproduction^26^. While these findings indicate functional interactions between E2 and leptin, primarily through the convergence of their central pathways^25,27^, these hormonal effects on peripheral regulators of energy metabolism are not well understood.

The gut microbiome, which is composed primarily of the bacteria in the intestinal tract and their metabolites, has profound effects on energy metabolism^28^. The gut microbiome aids in digestion and absorption of macro- and micro-nutrients from food^29^. Gut microbiota are also essential modulators of host immune homeostasis. Protective polysaccharides produced from the break-down of dietary fibers attenuate inflammation^30,31^. Gut microbiota can also synthesize and metabolize neurotransmitters and hormones to alter host physiology^32–34^. Furthermore, changes in body weight have been associated with changes in gut microbial diversity in humans and rodents^35^. For example, the transfer of gut microbiota from obese human or mice donors results in an obese phenotype in recipient mice^36–38^. Similarly, depletion of gut microbiota using antibiotics increases adiposity in mice^39^.

A variety of factors, including host genetics, diet, stress, and gonadal hormones can alter the gut microbiome^33,34,40–42^. Sex differences in gut microbiota have been reported in humans and rodents^41,43^. In a European population, higher levels of Bacteroidetes and Prevotella were observed in men compared to women^44^. Male mice exhibited higher abundances of Lachnospiraceae (phylum Firmicutes) and *Parabacteroides* spp. (phylum Bacteroidetes) and Proteobacteria than female mice^33,45^. Additionally, testosterone administration to female neonatal rats decreased gut microbial diversity during adulthood, and increased the ratio of the two most abundant phyla, Firmicutes and Bacteroidetes^40^. The hormone-dependent changes in gut microbiota were more robust compared to the diet-induced changes in these rats^40^. Ovariectomy also alters gut microbial diversity in adult mice^33,45,46^. While obesity and *ob/ob* genotype are associated with a reduction in Bacteroidetes/Firmicutes ratio in humans and male mice, respectively^47–50^, the effects of leptin on gut microbiota have not been studied in females. Since estrogens and leptin interact to profoundly affect female metabolic homeostasis^10,24^, it is important to understand the effects of leptin and its interaction with estrogens. Using a mouse model of obesity (*ob/ob*), the present study investigated if estradiol and leptin associate with longitudinal changes in gut microbiota and energy homeostasis in female mice on a HFD.

## Results

### Estradiol and ob/ob genotype altered weight gain and food intake in female mice on a HFD

**Figure 1:**
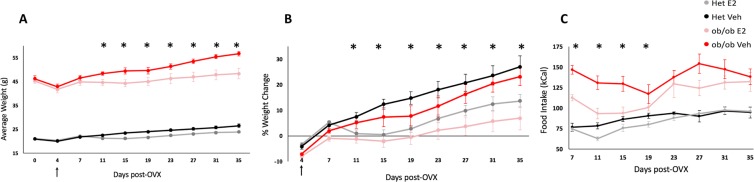
Estradiol and leptin alter weight gain and food intake in adult female *ob/ob* and lean control mice. (**A**) Average weight, (**B**) percent weight gain and (**C**) food intake (2 mice/cage) of heterozygote controls (Het) E2 (n = 13), Het Veh (n = 12), *ob/ob* E2 (n = 8) and *ob/ob* Veh (n = 7) with arrow indicating the start of high fat diet. The effects of genotype on average weight (**A**) and food intake (**C**) were present on all days. Days with effects of E2 are denoted by *(p < 0.05; ANOVA). Error bars indicate ± SEM.

Body weights were collected every four days from the four experimental groups: control E2 (n = 13), control Veh (n = 12), *ob/ob* E2 (n = 8), and *ob/ob* Veh (n = 8). Longitudinal analysis of weight change showed that E2 decreased weight gain (F = 15.67, p < 0.001; ANOVA) in female mice fed a HFD. E2 treatment attenuated weight gain from day 11 through the end of the study (Fig. 1A,B). Within-genotype comparison showed that E2 attenuated weight gain in Het mice from days 11–35, compared to Veh mice. For *ob/ob* mice, E2 reduced weight gain on days 11, 15 and 27–35, and showed strong trends on days 19 (p = 0.059) and 23 (p = 0.063). Because the Het and *ob/ob* mice had different weights at the beginning of the study, the % weight gain was also analyzed to remove the bias due to existing differences in weights prior to hormone and diet manipulation. Similar to the effects on body weight, E2 treatment reduced % weight gain from days 11–35. In particular, E2-treated Het mice gained less % weight than Veh Het mice on days 11–35 (Fig. 1B). Within the *ob/ob* mice, E2 decreased % weight gain on days 7–15 and 27–35.

Genotype also affected weight gain (F = 1119.79, p < 0.001; ANOVA) and % weight gain on longitudinal measures (F = 4.23, p = 0.047; ANOVA). *ob/ob* mice weighed more than control Het mice at all time points regardless of E2 treatment. At the start of the study, *ob/ob* mice weighed approximately twice that of the Het mice, which persisted throughout the study. Finally, there was an interaction of E2 and genotype on body weight (F = 4.21, p = 0.048; ANOVA), with E2-treated Het mice gaining more % weight than E2-treated *ob/ob* mice on days 4 and 7 (Fig. 1B). The Veh mice did not differ in % weight gain between the two genotypes.

E2 treatment reduced HFD consumption (F = 11.04, p = 0.002; ANOVA) from days 7 to 19 (Fig. 1C). Analysis within each genotype showed that E2 attenuated food intake in *ob/ob* mice, but not in Hets, on days 7, 11 and 15. Comparison between lean Hets and *ob/ob* mice showed that obesity profoundly increased food intake (F = 110.5, p < 0.001, ANOVA). Het mice consumed less calories than *ob/ob* mice throughout the study. Within the E2-treated groups, an increase in food intake in *ob/ob* groups compared to E2 Het mice was observed only at the beginning (days 7 and 11) and end (days 27, 31 and 35) of the study. There was an interaction between genotype and treatment on food intake (F = 5.52, p = 0.029; ANOVA). We also observed a decrease in food intake on day 19 in *ob/ob* Veh mice, but not other groups, following surgery on day 16 for removal of BrdU osmotic pumps.

### Estradiol and ob/ob genotype alter gut microbial diversity during HFD

**Figure 2:**
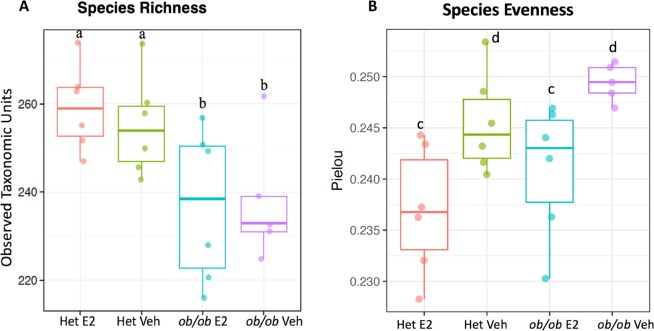
Estradiol and obesity reduce gut microbiota alpha diversity. (**A**) *ob/ob* genotype is associated with lower species richness as measured by Observed Taxonomic Units. (**B**) E2 treatment is associated with lower species evenness as measured by the Pielou’s evenness index. “a” and “b” indicate groups with different species richness while “c” and “d” indicate groups with different species evenness (p < 0.05, linear regression). Het E2 (n = 6), Het Veh (n = 6), *ob/ob* E2 (n = 6) and *ob/ob* Veh (n = 6).

To assess the impact of E2 treatment and obesity on α-diversity of the gut microbiota, 16S rDNA from fecal samples from *ob/ob* and Het mice were analyzed. The data set contained 137 samples after removing samples with less than 2,000 reads. The 16S rDNA targeted sequencing yielded 25,399 reads/sample on average (range 11,154–82,733). Clustering of these 16S sequence tags produced 473 OTUs at 97% similarity level. The identified OTUs belonged to 10 phyla, 42 families and 62 genera based on the Greengenes database. During HFD, *ob/ob* mice had a lower number of total identified OTUs than Het mice, indicating that *ob/ob* mice had lower species richness than control mice (Fig. 2A; p = 0.002, linear regression). In addition, E2 treatment was associated with lower species evenness in both genotypes, suggesting that E2-treated mice have a more heterogeneous distribution of gut microbial communities than Veh mice (Fig. 2B; p = 0.0008).

**Figure 3:**
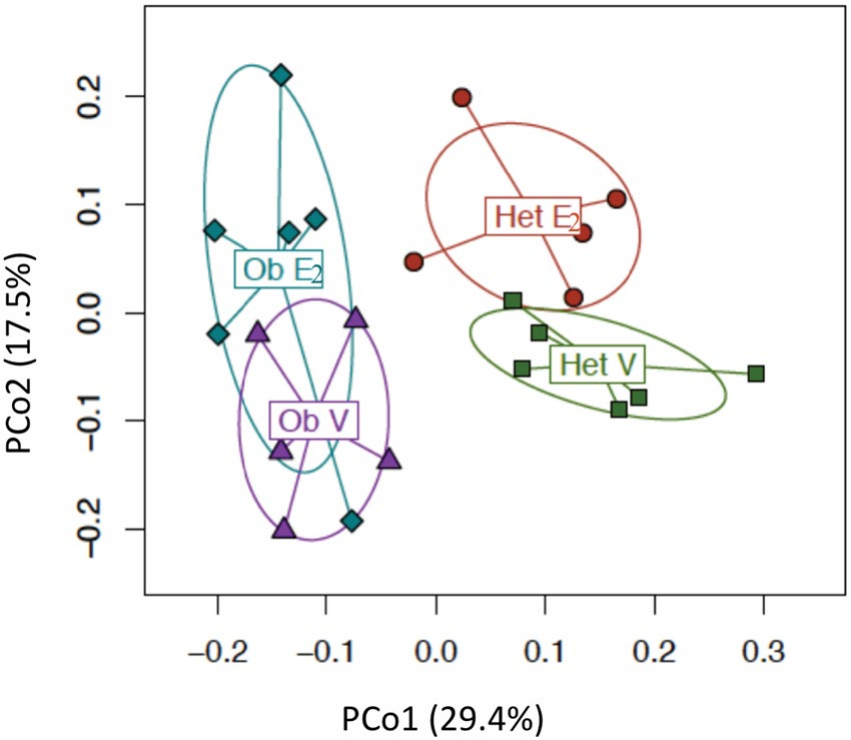
Gut microbial communities distinctly cluster as an effect of estradiol treatment and leptin. PCo1 and PCo2 clustering of each group. Bray-Curtis distance was used to calculate principal coordinates 1 and 2 and PERMANOVA, to calculate the association with leptin or E2 on aggregate data over all days. Ob E2 = E2*-*treated *ob/ob* (n = 6), Ob V = Vehicle *ob/ob* (n = 6), Het E2 = E2*-*treated Het (n = 6), and Het V = Vehicle Het mice (n = 6).

The ordination plot based on Bray-Curtis distance, which measures differences due to relative abundances and composition, showed distinct clustering of microbial communities from the four experimental groups (Fig. 3; p = 0.004, PERMANOVA). The first and second principal coordinates (PCo1 and PCo2) explain 29.4% and 17.5% of the variation in the microbial communities, respectively. Genotype, most likely through obesity, accounted for most of the difference observed as reflected by PCo1 (p = 4E-8, t-test, Fig. 3). There was also a clear effect of E2 treatment, represented by PCo2 (p = 0.001, t-test, Fig. 3).

Similar analyses were performed using Bray-Curtis distance measures on each day to characterize the temporal changes in gut microbiota. The effect of E2 on β-diversity was robust and continuously observed from the 2^nd^ week of OVX (days 15–35). The effect of genotype was also present on days 7,15, 23 and 35, and a trend was observed on the remaining two days (4 and 31) (Table 1).

**Table 1:** PERMANOVA P-values for individual days based on Bray-Curtis distance.

Day	Day 4	Day 7	Day 15	Day 23	Day 31	Day 35
Treatment	0.095	0.234	0.011^*^	0.044^*^	0.023^*^	0.022^*^
Genotype	0.081	0.004^*^	0.009^*^	0.001^*^	0.06	0.011^*^

^*^Indicate a p-value of <0.05.

### Estradiol treatment alters relative abundances of intestinal microbiota

**Figure 4:**
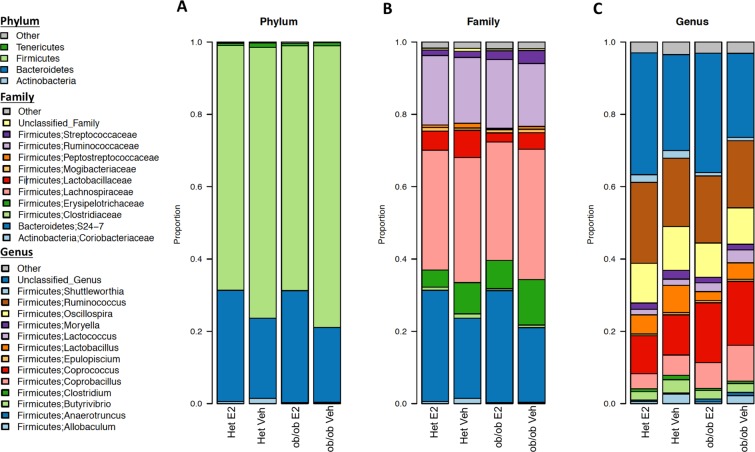
Gut microbiota associate with estradiol treatment and obesity at multiple taxa levels. Microbiota community structure at the (**A**) phylum, (**B**) family and (**C**) genus level, separated by estradiol treatment and *ob/ob* genotype. Data are shown as relative proportion of the taxa identified. Taxa with prevalence of >10% or with a maximum proportion of >0.2% were included. Het E2 (n = 6), Het Veh (n = 6), *ob/ob* E2 (n = 6), and *ob/ob* Veh mice (n = 6). An overdispersed Poisson regression was used to calculate associations of taxa with leptin and E2.

Gut microbial community composition was further analyzed on aggregate data from all days to identify the relative abundances at phylum, family and genus levels (Fig. 4). E2-mediated shifts in relative abundances was evident on many of these taxa levels. In all four experimental groups, the most prevalent phyla (>90%) were Firmicutes, Bacteroidetes, Actinobacteria and Tenericutes (Fig. 4A). The two most abundant families were S24-7 and Lachnospiraceae, within Bacteroidetes and Firmicutes, respectively (Fig. 4B). At the genus level, *Coprococcus* and *Ruminococcus* (both within phylum Firmicutes) were the most abundant (Fig. 4C).

**Figure 5:**
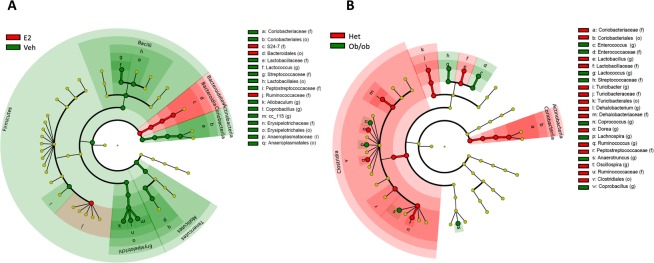
Estradiol and obesity associate with changes in the gut microbial composition. (**A**) Phyla and lower level taxa associated with estradiol (E2) or vehicle (Veh) treatment. (**B**) Phyla and lower level taxa associated with *ob/ob* or Het genotype. Het E2 (n = 6), Het Veh (n = 6), *ob/ob* E2 (n = 6) and *ob/ob* Veh (n = 6).

**Figure 6:**
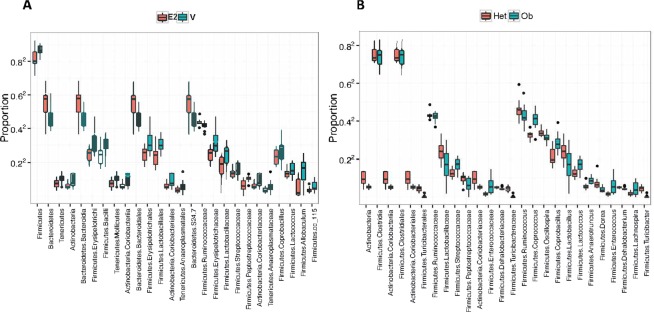
Gut microbiota associate with estradiol treatment and obesity at multiple taxa levels. Boxplots showing microbial taxa that are significantly altered by (**A**) Estradiol (E2) treatment and (**B**) genotype (overdispersed Poisson regression). Analysis was done on aggregate data from all 6 day points. Data are shown as relative proportion of the taxa identified. Taxa with prevalence of >10% or with a maximum proportion of >0.2% were included. E2*-*treated *ob/ob* (n = 6), E2*-*treated Het (n = 6), Veh *ob/ob* (n = 6), vehicle Het mice (n = 6).

To identify whether the differences in community structure were driven by differences in the relative abundances of particular microbial taxa, a differential abundance analysis based on overdispersed Poisson regression model was run on aggregate data to uncover the effects of treatment (Fig. 5A) and genotype (Fig. 5B). In the cladogram, the nodes in the first circle represent phyla, and the extending outer nodes in each level represent lower taxa within each phylum. A total of 26 taxa were differentially associated between E2 and Veh groups (Fig. 6A). Firmicutes were more associated with Veh treatment, while Bacteroidetes were associated with E2 treatment (Figs. 5A and 6A). Within Firmicutes, the class Erysipelotrichi, including *Allobaculum* and *Coprobacillus* spp., were more abundant in Veh than E2 mice. Similarly, the relative abundances of the class Bacilli, including its families Lactobacillaceae and Peptostreptococcaceae, were greater in Veh than E2 groups. Another family, Streptococcaceae, including *Lactococcus* spp., were also more associated with Veh mice. In contrast, the family Ruminococcaceae was more abundant in E2 mice. Within Bacteroidetes, the class Bacteroidia, and its lower taxa Bacteroidales and S24-7, were more abundant in E2 than Veh mice (Figs. 5A and 6A).

**Figure 7:**
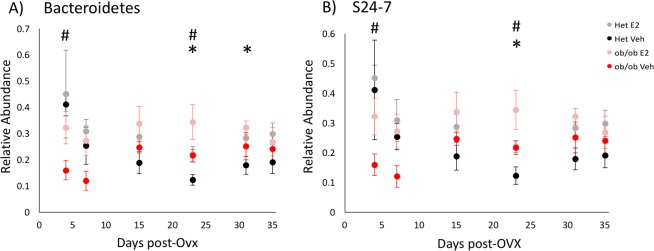
Estradiol and obesity associate with changes in the relative abundances of Bacteroidetes (phylum) and S24-7 (family) following the start of HFD. Relative abundances of the (**A**) the phylum Bacteroidetes and (**B**) its family S24-7 over time. *Indicates effects of E2 and # indicates effects of genotype (q < 0.1, overdispersed Poisson regression). Het E2 (n = 6), Het Veh (n = 6), *ob/ob* E2 (n = 6) and *ob/ob* Veh (n = 6). Error bars indicate ± SEM.

For the taxa that differed across groups as detected from the aggregated data, the effect of E2 treatment on relative abundances was analyzed for each day. A comprehensive list of the taxa, adjusted p-values (q-values) and fold changes between E2 and Veh mice for each day are provided in Supplemental Table 1. E2-treated mice resisted a decrease in relative abundances of Bacteroidetes compared to Veh mice on days 23 (q = 0.007) and 31 (q = 0.09) (Fig. 7A). A main driver of the shifts in Bacteroidetes was the family S24-7. E2-treated mice resisted a decrease in S24-7 abundances compared to Veh mice on day 23 (q = 0.015) (Fig. 7B).

**Figure 8:**
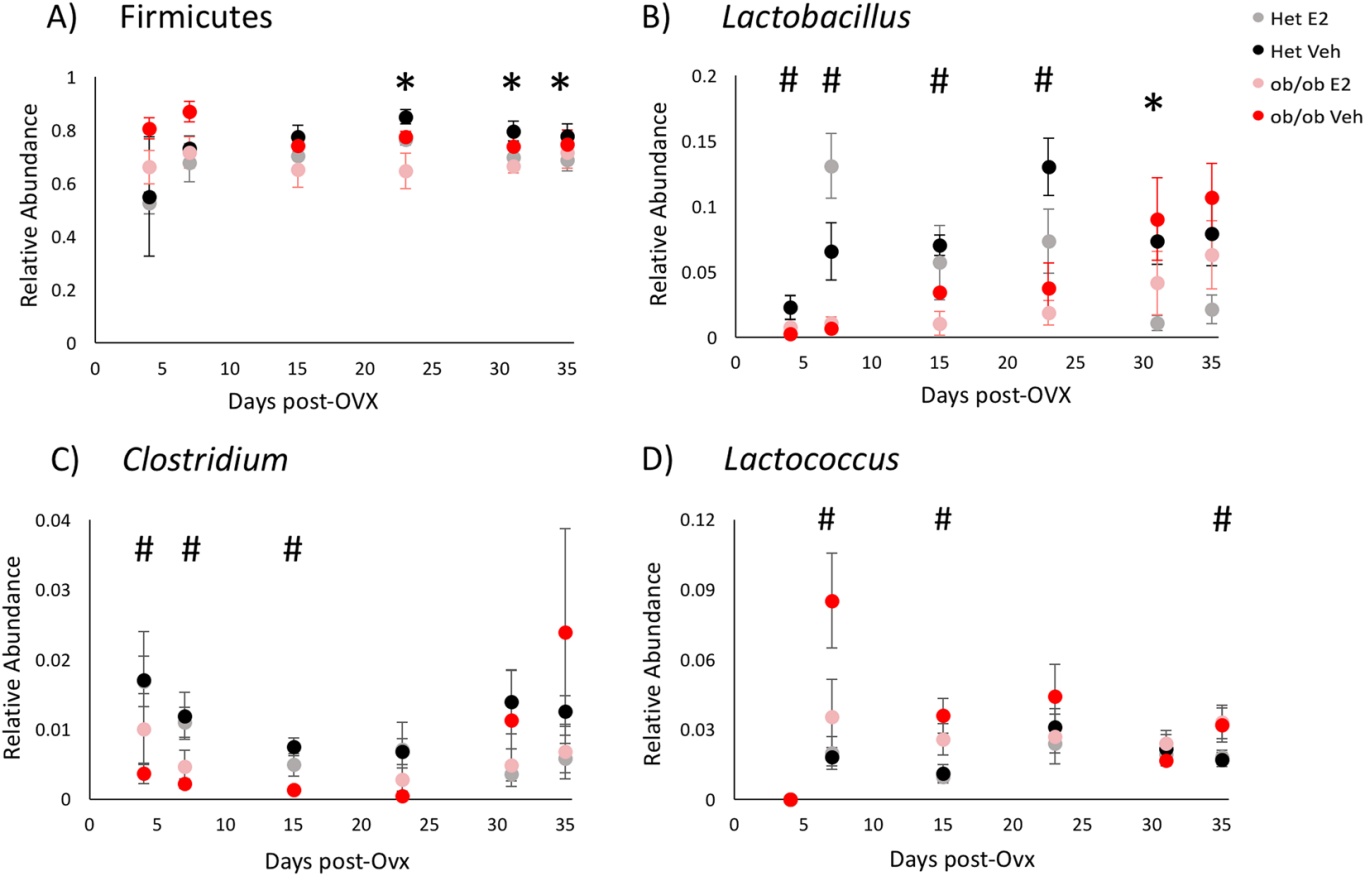
Estradiol and obesity associate with changes in the relative abundances of the phylum Firmicutes and its lower taxa. Relative abundances over time of the (**A**) phylum Firmicutes and genera (**B**) *Clostridium*, (**C**) *Lactobacillus* and (**D**) *Coprococcus*. *Indicates effects of E2 and # indicates effects of genotype (q < 0.1, overdispersed Poisson regression). Het E2 (n = 6), Het Veh (n = 6), *ob/ob* E2 (n = 6) and *ob/ob* Veh (n = 6). Error bars indicate ± SEM.

Compared to Veh mice, E2-treated animals resisted changes in relative abundances of the phylum Firmicutes, with lower relative abundances of Firmicutes on days 23 (q = 0.01), 31 (q = 0.06) and 35 (q = 0.04) compared to Veh mice (Fig. 8A). Within Firmicutes, *Lactobacillus* spp. showed a significant reduction on day 31 (q = 0.05) in E2-treated mice (Fig. 8B).

**Figure 9:**
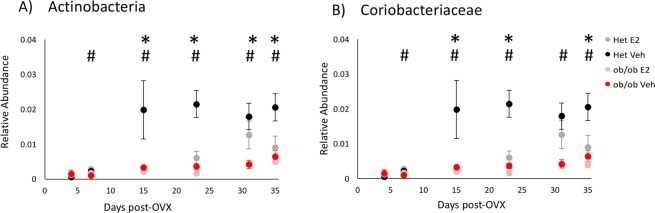
Estradiol and obesity resist increases in the relative abundances of the phylum Actinobacteria and its lower taxa. Relative abundances of the (**A**) phylum Actinobacteria and (**B**) family Coriobacteriaceae over time. *Indicates effects of E2 and # indicates effects of genotype (q < 0.1 overdispersed Poisson regression). Het E2 (n = 6), Het Veh (n = 6), *ob/ob* E2 (n = 6) and *ob/ob* Veh (n = 6). Error bars indicate ± SEM.

Estradiol also altered the relative abundance of the phylum Actinobacteria from days 15 to 35. On these days, E2-treated mice resisted an increase in the relative abundances of Actinobacteria compared to Veh mice (Fig. 9A). The E2-mediated effect on Actinobacteria was due mostly to the family Coriobacteriaceae which resisted this increase also on days 15 (q = 0.001), 23 (q < 0.001) and 35 (q = 0.03) (Fig. 9B).

### Obesity is associated with changes in relative abundances of gut microbiota

As the gut microbial communities of the female mice clustered separately based on obesity (in *ob/ob* genotype) (Fig. 3), we further analyzed the effects of obesity on relative abundances at the taxa levels (Fig. 5B). A total of 26 identified taxa showed a differential association between *ob/ob* and Het mice (Fig. 6B). Comparison of the gut microbial communities between genotypes revealed that Firmicutes, including its families Lactobacillaceae, Turibacteraceae, Peptostreptococcaceae, Dehalobacteriaceae, and Ruminococcaceae were more abundant in Het mice than *ob/ob mice*. At the genus level, *Lactobacillus, Turibacter, Dehalobacterium, Dorea, Ruminococcus*, and *Oscillospira* were more abundant in Het controls than *ob/ob*. Similarly, the phylum Actinobacteria and its family Coriobacteriaceae showed a greater abundance in control Het mice. In contrast, *Enterococcus*, *Coprococcus*, *Lachnospira*, *Anaerotruncus* and *Coprobacillus* spp. of the phylum Firmicutes were more abundant in *ob/ob* mice compared to Het mice (Figs. 5B and 6B).

Differential abundance analysis across days showed that genotype affected the relative abundances of multiple taxa (Supplemental Table 2). Leptin was associated with increases in the relative abundances of Bacteroidetes and its family S24-7 in Het mice at the beginning (day 4; q = 0.04), but by day 23 (after 19 days on HFD; q = 0.03), these were lower than in *ob/ob* mice (Fig. 7). Within Firmicutes*, Clostridium*, *Lactobacillus* and *Lactococcus* spp. were altered by genotype (Fig. 8). *Lactobacillus and Clostridium* spp. were more abundant in Het mice. The relative abundance of *Lactobacillus* increased in Het mice compared to *ob/ob* mice on days 4 (q = 0.01), 7 (q < 0.001) and 23 (q = 0.001) (Fig. 8B). Similarly, *Clostridium* was higher in Het mice on days 4 (q = 0.04), 7 (q = 0.01) and 15 (q < 0.001) than *ob/ob* mice (Fig. 8C). In contrast, *Lactococcus* was more abundant in *ob/ob* mice on days 7 (q = 0.01), 15 (q = 0.002) and 35 (q = 0.007) (Fig. 8D).

Obesity was also associated with altered relative abundance of the phylum Actinobacteria over time. Compared to *ob/ob* mice, Het mice had an increase in the relative abundance of Actinobacteria from days 7 to 35 (Fig. 9A). This leptin-dependent increase in Actinobacteria in Hets was due mostly to Coriobacteriaceae (family), also from days 7 to 35 (Fig. 9B), suggesting this microbial family is influenced by leptin or obesity status during HFD.

A subset of microbial taxa was affected by both E2 and leptin. In particular, Streptococcaceae, *Lactococcus* and *Coprobacillus* were decreased, whereas Ruminococcaceae were increased by E2 and leptin. In addition, these two hormones exerted opposing effects on other families, such that E2 decreased, and leptin (in Het controls) increased, abundances of Coriobacteriaceae, Lactobacillaceae and Peptostreptococcaceae.

## Discussion

Mounting evidence indicates profound effects of estrogens, leptin and gut microbiota on energy homeostasis^10,25,51,52^. The present study investigated if estradiol and leptin-mediated energy regulation is associated with temporal changes in gut microbiota of females. Using leptin-deficient *ob/ob* female mice fed a HFD, we investigated the effects of estradiol and obesity (due to leptin deficiency) on body weight, energy intake, and gut microbiota in the present study. We found that E2 treatment decreases food intake and protects against HFD-induced weight gain in both *ob/ob* (obese) and Het (lean) females. *ob/ob* mice had increased HFD intake and greater body weight, compared to the Het controls. The differences in food intake and body weight between the two genotypes are reflected in their changes in gut microbiota. Both E2 treatment and genotype were associated with altered gut microbiota diversity. o*b/ob* female mice exhibited lower species richness, which supports previous studies that link obesity with a reduced gut microbiota diversity in humans and male mice^53–55^. Interestingly, E2 treatment was associated with lower species evenness compared to Veh mice. At the phylum level, E2 treatment slowed down a HFD-induced decrease in Bacteroidetes and increases in Firmicutes and Actinobacteria compared to Veh controls. Leptin also was associated with changes in Bacteroidetes and Firmicutes and a profound increase in Actinobacteria. Many taxa were also associated with both leptin and E2. These findings suggest that E2 and leptin can act independently, or interact together, to modulate gut microbiota to mediate energy regulation during HFD intake in females.

We and others have shown that E2 protects female mice from HFD-induced obesity^10,11,13,56–59^. Estrogens exert protective effects by acting directly on brain^10–12,60^, pancreas^61^, liver^56,57,59,62^, adipose tissue^59^, and muscle^63,64^ to regulate energy production and utilization. Moreover, long-term E2 treatment improves glucose tolerance and insulin sensitivity and attenuates lipid synthesis in liver in *ob/ob* female mice^65^. The present findings suggest that the modulation of gut microbiota is another mechanism by which E2 mediates energy homeostasis. While E2 was associated with a decrease in the gut microbial evenness in the current study, an increase in microbial diversity has been reported in cycling rats and E2-treated female mice^40,66^. These differences across studies could be due to fluctuating estrogens and other ovarian hormones in the cycling rats and/or species differences^40^, and a much higher dose of estradiol (2.5 mg/day, consistent with levels at pregnancy) used in the mice^66^. Alternatively, while a lower microbial diversity is usually associated with obesity and metabolic disorders^67,68^, a change in relative abundance without any changes in microbial richness or evenness can also alter microbial homeostasis^47^.

E2 attenuated a longitudinal shift in the two major phyla Bacteroidetes and Firmicutes, compared to the Veh controls. In particular, the order Bacteroidales, and its families S24-7 and Ruminococcaceae were positively associated with E2 treatment, whereas *Allobaculum* spp. were negatively associated. Many microbes that belong to S24-7 produce short chain fatty acids (SCFA; fermented products of dietary fibers) that protect against inflammation^69,70^. In particular, SCFA, including butyrate, maintain a low pH in the gastrointestinal tract and aid in nutrient absorption and pathogen inactivation^71^. Interestingly, S24-7 is downregulated in inflammatory conditions, including Crohn’s disease, colitis and type I diabetes^72–74^. While the current findings suggest that E2 provides protection against diet-induced metabolic disorders in females by maintaining healthy levels of microbes belonging to S24-7, it will be important in future studies to selectively manipulate these microbes to test their functions in energy regulation.

In the present study, E2 was associated with changes in Firmicutes, which is generally associated with obesity in rodents and humans^75^. Studies in males have shown that a high caloric diet profoundly affects Firmicutes^36,76,77^. Consistent with these findings in males, in the present study, Veh female mice on a HFD gained weight and had an increase in relative abundances of Firmicutes. However, E2-treated mice resisted this HFD-induced increase, suggesting that modulation of these microbes contributes to the regulation of energy homeostasis by estrogens. Firmicutes contain more OTUs that are efficient energy producers than Bacteroidetes, leading to increased calorie absorption and weight gain^52^. Additionally, some members of Firmicutes promote lipid droplet formation increasing fatty acid absorption and weight gain^78^. In the current study, E2 was associated with changes in relative abundances of Firmicutes in both Het and *ob/ob* mice, suggesting that E2 affects this phylum independent of body weight and leptin levels.

The obese leptin-deficient (*ob/ob*) mice had less diverse microbiota as observed by species richness, suggesting that leptin is required for an enriched microbial ecosystem. In support, obesity is associated with a decreased microbial diversity in humans^53^. Furthermore, a Danish study on men and women found that higher levels of leptin in obese populations were associated with a lower richness of the gut microbial communities, suggesting that optimum levels of leptin are associated with metabolic health and a more diverse gut microbiota^54^. Taken together, the present results suggest that changes in leptin levels are associated with disruptions in metabolic and microbial homeostasis.

While *ob/ob* mice weighed almost twice that of Het mice in the beginning, the percent weight gain was greater in Veh Hets compared to Veh *ob/ob* mice. Interestingly, the relative abundance of the phylum Actinobacteria was greater in Hets compared to *ob/ob* mice, primarily due to an increase in its family Coriobacteraceae. Actinobacteria is increased in obese humans and is associated with ulcerative colitis^53,68^. More than two-thirds of the obesity-related human gut microbes belong to Actinobacteria^53,79^. In particular, Coriobacteriaceae have been positively associated with increased cholesterol absorption in hamsters and obese humans^80,81^. A profound increase in the relative abundance of Coriobacteriaceae in Veh Het mice, the group that showed the highest percent weight gain, but not in E2 Hets, suggests this OTU is associated with HFD-induced weight gain.

Estrogens elicit many effects on physiology and behavior by binding to their intracellular and membrane receptors^82–85^. While estrogen receptor-α (ERα) and ERβ have been implicated in the effects of estrogens on metabolism^86–88^, ERα appears to be the primary contributor to energy balance^86,89–91^. ERα knock-out mice exhibit increased visceral adiposity, impaired glucose tolerance and elevated insulin levels^91^. Furthermore, systemic activation of ERα, but not ERβ, decreases food intake and body weight in female rats^90^. While the effects of ERα on gut microbiota have not been investigated directly, a study using ERβ knock-out female mice suggests that ERβ influences gut microbiota in a diet-specific manner^92^. In support of this finding, ERβ is expressed in human and mouse colon epithelium^93,94^. Alternatively, given that estrogens reduce food intake^10^, it is also possible that E2 influences gut microbiota by altering nutrition availability.

Estrogens may also act as direct substrates for gut microbiota. For example, microbes with β-glucuronidase and β-glucosidase enzymes, including *Lactobacillus, Bifidobacterium*, and *Clostridium* spp., convert inactive estrogens into their active forms through deconjugation^95–97^. In addition, estrogens may alter metabolism and immune responses through direct actions on gut microbial metabolites. For example, E2 treatment protects against HFD-induced metabolic disorders by blocking the activation of lipopolysaccharides (LPS), the endotoxins produced by gram-negative microbes^98–100^. Additionally, E2 upregulates intestinal alkaline phosphatase, a protective enzyme in the gut epithelium that functions by attenuating pro-inflammatory signals^45^. These studies, taken together with the present findings, suggest that estrogens can alter gut microbiota through multiple direct and indirect mechanisms to protect from metabolic disorders.

The mechanisms by which leptin affects gut microbiota are not well understood. Intestinal epithelium expresses leptin receptors (LepR), but ablation of these receptors does not alter gut microbiota or body weight in male mice^101^. In females, leptin may act in concert with ERß in the gut epithelium^93,94^. While genotype effects may be primarily dependent on leptin, it is important to note that *ob/ob* mice are obese and Het mice are lean. Obesity is an independent modulator of gut microbiota. Obese and lean twins exhibit differences in gut microbial diversity^53^, and the obese phenotype can be transferred to lean recipients through the transplant of gut microbiota from obese donors^36,37^. Alternately, HFD and obesity directly alter the host transcriptome and epigenome by differentially activating enhancers and promoters in the intestinal epithelium^102^. In addition, female *ob/ob* mice have reduced estrogen levels and are sterile, and thus have altered hormone-dependent development^26,103^. These findings suggest that leptin, acting directly, or indirectly via alteration of obesity status, affects host physiology, including gut microbial homeostasis. In future studies, it will be important to test the effects of leptin administration on the gut microbiota in HFD-fed ovariectomized *ob/ob* mice.

This study provides further insights into the potential roles of E2 and leptin in HFD-induced obesity and gut microbiota. While the identification of taxa that differ between treatments is the first step, their potential actions on energy metabolism will need to be explored in functional studies that manipulate the microbiota using fecal microbiota transplant, selective administration, or depletion of the target microbes. Given that many metabolic diseases are characterized by disturbances in the gut microbiome, investigating how estradiol and leptin-dependent energy regulation associates with disruption in microbial homeostasis provides a foundation for future functional studies on the role of these hormones and the gut microbiome in metabolic disorders in women.

## Materials and Methods

### Animals

Seven week-old lean heterozygote (Het) and obese *ob/ob* (leptin-deficient) mice (24 mice/genotype) were purchased from Jackson Laboratory (Bar Harbor, Maine), kept on a 12:12 light:dark cycle and allowed to acclimate at the Wellesley College animal facility for a week. Het mice, which have a metabolic phenotype similar to wildtype mice as evidenced by normal serum glucose and insulin, body temperature, and energy expenditure^19^, were used as controls^21^ to account for genetic background. While phenotypically very similar, Het mice have lower leptin levels than wildtype mice^104^. All mice were ovariectomized (OVX) and subcutaneously implanted with a silastic capsule containing 17 $\beta$-Estradiol (E2, #E8875, Sigma; 50 µg in 25 µl of 5% ethanol/sesame oil) or Veh (5% ethanol/sesame oil) resulting in the following four experimental groups: control E2, 2) control Veh, 3) *ob/ob* E2, and 4) *ob/ob* Veh. Following surgery, mice were pair-housed with a mouse of the same genotype and treatment.

As part of another study to identify newborn cells, on day 7 mice underwent intracranial surgery for the implantation of an intraventricular cannula attached to a subcutaneous osmotic pump filled with bromodeoxyuridine. The osmotic pumps were removed after 10 days. On day 35, animals received an intraperitoneal injection of leptin (5 mg/kg) 45 minutes prior to being euthanized for assessment of acute leptin response in the brain, unrelated to the present study. While possible, it is unlikely that leptin administration 45 minutes prior to sacrifice would have effects on gut microbiota. All procedures were approved by the Institutional Animal Care and Use Committee of Wellesley College and performed in accordance with National Institutes of Health Animal Care and Use Guidelines.

### Food intake and body weight measurements and fecal sample collection

Following OVX (day 0), all mice were maintained on a standard diet for three days (13.5% calories from fat, Purina, #5001) before switching to a HFD (58.4% calories from fat, Teklad, #03584) on day 4 and maintained on a HFD for the remainder of the study. Throughout the experiment, food intake and body weights were recorded every four days from heterozygote control (Het) E2 (n = 13), Het Veh (n = 12), *ob/ob* E2 (n = 8) and *ob/ob* Veh (n = 7) mice. Food intake was calculated per cage, from a total of Het E2 (n = 6), Het Veh (n = 6), *ob/ob* E2 (n = 6) and *ob/ob* Veh (n = 6) cages, with each cage containing two mice of the same treatment. For cages in which one mouse died, the amount of food eaten by the remaining mouse was doubled to match with cages containing two mice. Similarly, for microbiota analysis, fecal samples from each cage was counted as n = 1, resulting in Het E2 (n = 6), Het Veh (n = 6), *ob/ob* E2 (n = 6) and *ob/ob* Veh (n = 6). Fecal samples were collected on days 4, 7, 15, 23, 31, and 35 and immediately stored at −80 °C.

### DNA extraction from fecal samples, 16S rDNA sequencing and bioinformatics processing

DNA was extracted from fecal samples using a MO BIO PowerSoil DNA Isolation Kit (Valencia, CA) with minor adjustments to the manufacturer’s protocol. A 5-minute incubation with the elution buffer before centrifugation was added to increase the DNA yield. The quality and quantity of the DNA samples were measured using Nanodrop (Thermo Scientific, Waltham, MA). The samples were stored at −20 °C until sequencing.

The V3-V4 region of the 16S rDNA was amplified using the following universal 16S rDNA primers: forward 341F (5′-CCTACGGGAGGCAGCAG-3′) and reverse 806R (5′-GGACTACHVGGGTWTCTAAT-3′) with sequence adapters on both primers and sample-specific Golay barcodes on the reverse primer^105^. The PCR products were quantified by PicoGreen (Invitrogen, Carlsbad, CA) using a plate reader. After quantification, amplicons were pooled in equal concentrations, cleaned up using UltraClean PCR Clean-Up Kit (MO BIO, Carlsbad, CA), and again quantified using the Qubit (Invitrogen, Carlsbad, CA). The pooled samples were then sequenced using paired-end v2 chemistry using Illumina Miseq sequencing technology (Illumina, San Diego, CA) at the Microbiome Core, Mayo Clinic (Rochester, Minnesota).

Paired R1 and R2 sequence reads were then processed via the *hybrid-denovo* bioinformatics pipeline, which clustered a mixture of good-quality paired-end and single-end reads into operational taxonomic units (OTUs) at 97% similarity level^106^. OTUs were assigned taxonomy using the RDP classifier trained on the Greengenes database (v13.5)^107^. A phylogenetic tree based on FastTree algorithm was constructed based on the OTU representative sequences^108^. Singleton OTUs as well as samples with less than 2,000 reads were removed from downstream analysis.

### Statistical analysis

#### Food intake and body weight data analysis

Repeated measures ANOVA (SPSS, v.24) was performed to examine the effects of treatment and genotype on food intake and body weight over time. After a main effect was confirmed, ANOVA without corrections was performed on measures from each day to identify the days with effects of one or both variables. One–way ANOVAs were then conducted to identify differences between specific groups when effects of treatment or genotype were present. Differences were considered statistically significant at p < 0.05.

#### 16S rDNA sequence analysis

Analyses were first performed on the aggregated data, in which sequence reads from each mouse across all days were aggregated. To study specific longitudinal trends, stratified analyses on individual days were also performed if needed.

#### Diversity analysis

Both α-diversity and β-diversity were analyzed on the rarefied OTU data. α-diversity (within-sample diversity) reflects species richness and evenness within the microbial populations. Two representative α-diversity measures were investigated: the observed number of OTUs, an index of the species richness, and the Pielou’s evenness index^109^. A multiple linear regression model (“lm” function in R) was used to test the association between α-diversity (outcome) and treatment/genotype (covariates, both included in the model).

β-diversity (between-sample diversity) reflects the shared diversity between bacterial populations in terms of ecological distances; pair-wise distance measure allows quantification of the overall compositional difference between samples. Different β-diversity measures provide distinctive views of the community structure. The β-diversity measures were calculated using Bray-Curtis dissimilarity, which measures differences in bacterial composition based on taxa abundances (“vegdist” function in the R “vegan” package, v2.4-3). To test the association between β-diversity measures and treatment or genotype, we used PERMANOVA (999 permutations, “adonis” function in the R “vegan” package, v2.4-3) when adjusting the effect of the other covariate. Ordination plots were generated using principal coordinate analysis (PCoA) on the distance matrix (“cmdscale” function in R)^110^.

#### Taxa analysis

Differential abundance analyses were performed at the phylum, class, order, family and genus levels. Taxa with prevalence less than 10% or with a maximum proportion less than 0.2% were excluded from analysis to reduce the number of tests. An overdispersed Poisson model was fitted to the taxa counts with treatment and genotype as covariates (“glm” function in R)^111^. Wald test was used to assess significance. To account for variable sequencing depths, the GMPR size factor was estimated and used as an offset (log scale) in the regression model^112^. False discovery rate (FDR) control (B-H procedure, ‘p.adjust’ in R) was used to correct for multiple testing at each taxonomical level, and FDR-adjusted p-values (q-values) < 0.1 were considered significant^113^. The differential taxa were visualized on a cladogram using GraPhlAn^114^. All statistical analyses were performed in R (v. 3.3.2, R Development Core Team).

## Supplementary information

Supplementary Information.

Supplementary Table 1.

Supplementary Table 2.
