# Author Correction: Estradiol and high fat diet associate with changes in gut microbiota in female *ob/ob* mice

**DOI:** 10.1038/s41598-021-87104-2

**Published:** 2021-04-12

**Authors:** Kalpana D. Acharya, Xing Gao, Elizabeth P. Bless, Jun Chen, Marc J. Tetel

**Affiliations:** 1 Neuroscience Department, Wellesley College, Wellesley, MA 02481 USA; 2 Department of Health Sciences Research & Center for Individualized Medicine, Mayo Clinic, Rochester, MN 55905 USA

Correction to: *Scientific Reports* 10.1038/s41598-019-56723-1, published online 27 December 2019

The original version of this Article contained an error in the legend of Figure 8.

“Estradiol and obesity associate with changes in the relative abundances of the phylum Firmicutes and its lower taxa. Relative abundances over time of the (**A**) phylum Firmicutes and genera (**B**) *Clostridium*, (**C**) *Lactobacillus* and (**D**) *Coprococcus*. *Indicates effects of E2 and # indicates effects of genotype (q < 0.1, overdispersed Poisson regression). Het E2 (n = 6), Het Veh (n = 6), *ob/ob* E2 (n = 6) and *ob/ob* Veh (n = 6). Error bars indicate ± SEM.”

now reads:

“Estradiol and obesity associate with changes in the relative abundances of the phylum Firmicutes and its lower taxa. Relative abundances over time of the (**A**) phylum Firmicutes and genera (**B**) *Lactobacillus*, (**C**) *Clostridium*, and (**D**) *Lactococcus*. *Indicates effects of E2 and # indicates effects of genotype (q < 0.1, overdispersed Poisson regression). Het E2 (n = 6), Het Veh (n = 6), *ob/ob* E2 (n = 6) and *ob/ob* Veh (n = 6). Error bars indicate ± SEM.”

The original Article has been corrected.

